# Spinal cord injury in an adult patient with thoracic butterfly vertebra: a case report and review of the literature

**DOI:** 10.1186/s12891-020-03433-9

**Published:** 2020-06-22

**Authors:** Ai-Bing Huang, Meng Bai, Huan Liu, Zhi-Yong Zhou, Jia-Xu Wei

**Affiliations:** 1 Department of Orthopedics, The Hospital Affiliated with the Medical School of Yangzhou University (Taizhou People’s Hospital), No. 366, Taihu Road, Taizhou, 225300 Jiangsu Province China; 2 Postgraduate School, Dalian Medical University, Dalian, 116000 Liaoning China

**Keywords:** Spinal cord injury, Butterfly vertebra, Congenital, Asymptomatic, Case report

## Abstract

**Background:**

Butterfly vertebrae are a rare congenital vertebral anomaly. An overlap of this spinal anomaly with other diseases has been reported. However, to the authors’ knowledge, the coexistence of butterfly vertebrae and spinal cord injury has not been reported in the literature.

**Case presentation:**

A 42-year-old male was admitted to our emergency department after a motor vehicle accident. His complaint was back pain, and he was unable to move both lower limbs. Upon physical examination, the patient was not ambulatory. Sensory examination revealed the absence of sensation below the T12 level. The strength of the bilateral lower limbs was grade 0. The patient received a radiographic evaluation. The initial diagnosis was T11 fracture with complete paraplegia of the lower limbs. Magnetic resonance imaging (MRI) was then performed. Sagittal MRI demonstrated an isointense lesion on T1-weighted imaging and a high-signal spindle-like lesion on T2-weighted imaging of the spinal cord adjacent to the T11 vertebra. The fat-suppressed sequence also revealed hyperintensities of the cord. There was no evidence of acute injury of the T11 vertebral body except for cuneiform anterior wedging. The patient was ultimately diagnosed with complete paraplegia with a T11 butterfly vertebra. He underwent urgent posterior decompressive and fixation surgery from T10 to T12. His postoperative recovery was uneventful.

**Conclusions:**

The coexistence of a butterfly vertebra with spinal cord injury was reported for the first time. Although butterfly vertebrae may be incidentally detected, it is important to be familiar with their radiographic features to distinguish them from fractures.

## Background

Congenital vertebral anomalies represent a spectrum of spinal deformities due to developmental defects that produce an imbalance in the longitudinal growth of the spine. Based on embryonic development, two basic types of anomalies, namely, failure of formation and failure of segmentation, have been recognized [1]. A butterfly vertebra is a rare formation defect resulting from failure of fusion of the two chondrification centers during fetal development [2].

Butterfly vertebrae are often asymptomatic and incidentally detected [2]. The overlap of this spinal anomaly with other syndromes has been well illustrated [3]. Moreover, the condition has occasionally been reported to be associated with other diseases, such as disc herniation and ankylosing spondylitis [4, 5]. However, to the authors’ knowledge, the co-occurrence of butterfly vertebrae and spinal cord injury is an unusual event that has not been reported in the literature. Here, we describe a rare case of spinal cord injury in an adult patient with a thoracic butterfly vertebra.

## Case presentation

A 42-year-old male was admitted to our emergency department 3 h after a motor vehicle accident. He was thrown out of the car because he was not wearing a seatbelt. His complaint was back pain, and he was unable to move both lower limbs. His medical history was unremarkable.

**Fig. 1:**
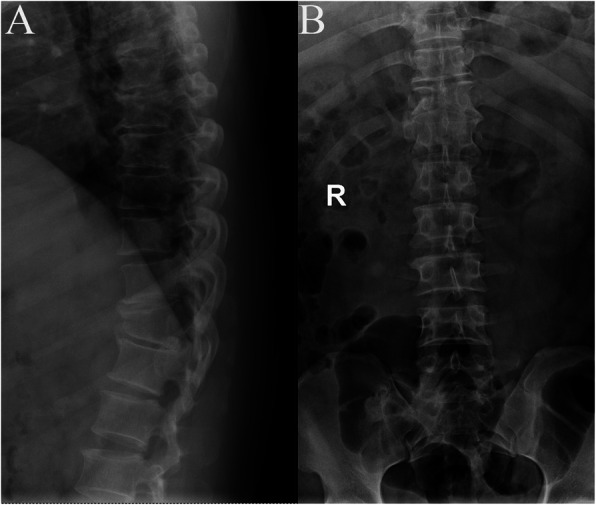
Plain radiograph of the thoracic vertebrae (**a**, lateral view) shows anterior wedging of T11, which could be easily confused for a compression fracture. An anteroposterior view (**b**) shows a symmetrical defect with corticated margins in the T11 vertebral body

Upon physical examination, the patient was not ambulatory. Sensory examination revealed the absence of sensation below the T12 level. The strength of the bilateral lower limbs was grade 0. The patient underwent radiographic evaluation, which showed wedging of the T11 vertebra suggestive of a compression fracture (Fig. 1a). An initial diagnosis of T11 fracture with complete paraplegia of the lower limbs was made based on these findings.

**Fig. 2:**
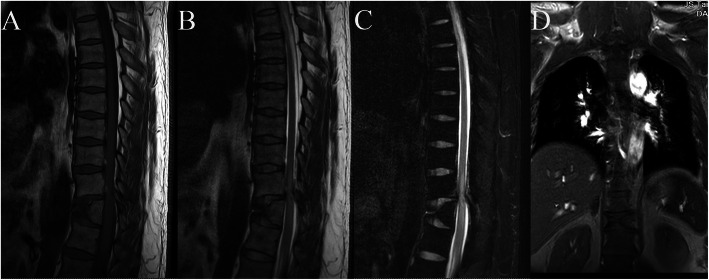
Magnetic resonance imaging (MRI) of the spine after injury. T1-weighted MRI (**a**) shows normalized signal intensities of the spinal cord. T2-weighted (**b**) and fat-suppressed MRI sequences (**c**) show a high-signal lesion in the spinal cord adjacent to the T11 vertebra. A coronal image (**d**) shows the presence of a butterfly vertebra at the T11 level. There was no signal change in the vertebral bodies or intervertebral discs

Magnetic resonance imaging (MRI) was further conducted to evaluate the spinal cord. Sagittal MRI demonstrated an isointense lesion on T1-weighted images (Fig. 2a) and a high-signal spindle-like lesion on T2-weighted images of the spinal cord adjacent to the T11 vertebra (Fig. 2b). The fat-suppressed sequence also revealed hyperintensities of the cord (Fig. 2c). Injury to the posterior ligament complex (PLC) was detected by MRI, and the integrity of the complex was considered indeterminate. There was no evidence of acute injury of the T11 vertebral body except for cuneiform anterior wedging. Then, we reevaluated the anteroposterior view (Fig. 1b), and the appearance of the vertebral body defect was symmetrical with corticated margins, suggesting a T11 butterfly vertebra. Finally, a primary diagnosis of complete paraplegia with a T11 butterfly vertebra was made. According to the AOSpine classification, the fracture type was B2 N4.

**Fig. 3:**
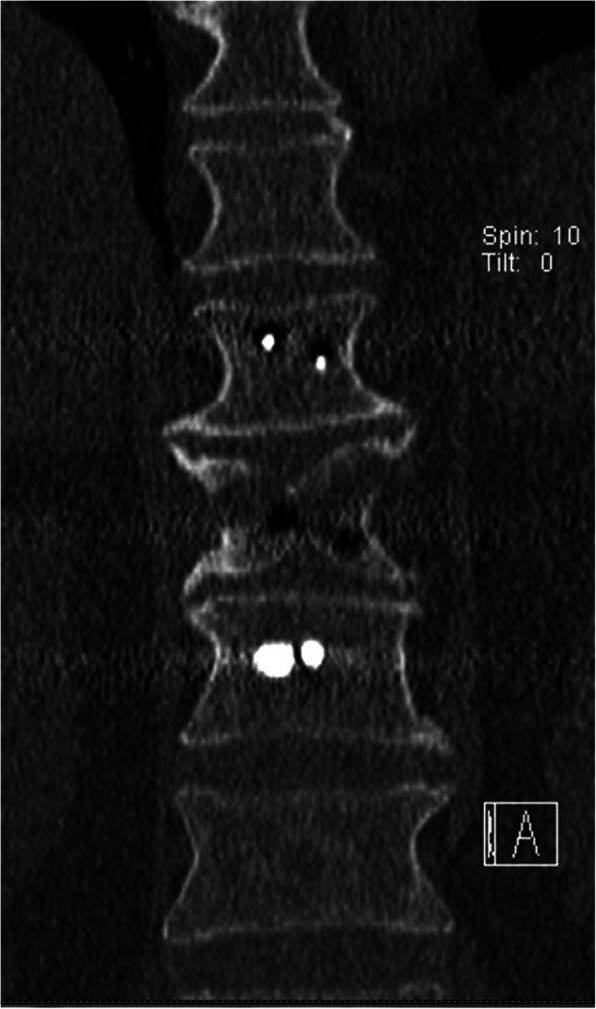
A postoperative computerized tomographic scan shows a symmetrical defect with corticated margins in the vertebral body, confirming the presence of a butterfly vertebra

**Fig. 4:**
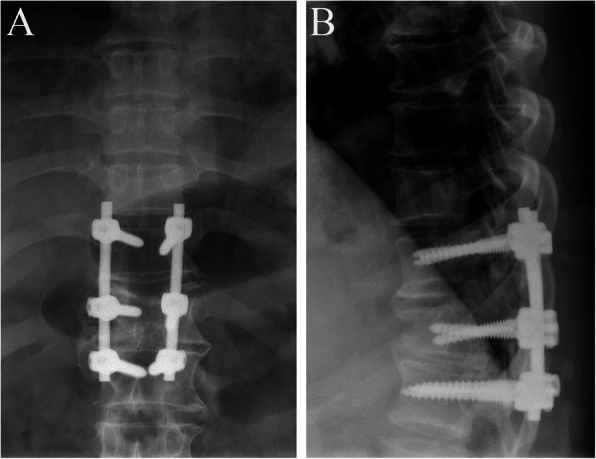
After 12 months of follow-up, radiographic images show no evidence of implant migration or disruption

The patient underwent urgent posterior decompressive and fixation surgery from T10 to T12. The disruption of the PLC was confirmed during the operation. The patient’s postoperative recovery was uneventful. There was no wound infection, and no complications were observed. Postoperative computed tomography (CT) examinations (Fig. 3) were performed, confirming the presence of a T11 butterfly vertebra consisting of two lateral hemivertebrae. The patient was discharged after 25 days of hospitalization and was transferred to a rehabilitation center. After 12 months of follow-up, the patient did not show any improvement in neurological status (American Spinal Injury Association, ASIA, grade A), and radiographic images revealed no evidence of implant migration or disruption (Fig. 4).

## Discussion and conclusion

Butterfly vertebrae are rare congenital malformations that resemble butterfly wings on an anterior-posterior radiographic view. This uncommon deformity is usually reported as an incidental finding and can be asymptomatic throughout life. It can occur as an isolated anomaly or in association with other congenital syndromes. However, the coexistence of this anomaly with other diseases has been reported in the literature. Delgado et al. [6] reported a 41-year-old man with an L3 butterfly vertebra who was found to have concurrent lumbar disc protrusion at a different intervertebral level. Moreover, Cho et al. [4] treated a novel case involving a butterfly vertebra that overlapped with symptomatic disc herniation of the sagittal cleft. Recently, Qian et al. [5] described 4 cases of co-occurrence between congenital spinal deformities and ankylosing spondylitis. Among these cases, two patients were classified as having butterfly vertebrae. The authors pointed out that it was difficult to make an early differential diagnosis when the two diseases coincided.

Butterfly vertebrae present a wedge-shaped configuration on a lateral view and can be easily confused with a compression fracture [7–11]. In an anteroposterior view, the butterfly vertebra is split into two hemivertebrae, and the pedicles appear divergent. Satpathy et al. [10] pointed out that CT or MRI scans are indicated in doubtful cases, as they may demonstrate a sagittal cleft defect with sclerotic margins. In addition, the disc of the defective vertebra may show density irregularities continuous with the discs above and below the butterfly vertebra. Awareness of these imaging features is critical for making a correct diagnosis [8]. In the present case, the initial diagnosis was a T11 fracture with complete paraplegia. The characteristic MRI findings described above led us to correct our final diagnosis. Several previous reports have also provided insight into this clinical entity. Garcia et al. [11] described two patients who had injuries of the dorsal spine with vertebral wedging. Initially, a compression wedge fracture was diagnosed, but after careful study of the radiographs, both cases were confirmed to be butterfly vertebrae. This finding suggests that this rare congenital anomaly may be mistaken for a wedge fracture unless the anteroposterior view is correctly assessed. Guo et al. [7] reported on a 4-year-old boy with a T1 butterfly vertebra who suffered from a fall at home and was initially misdiagnosed with a burst fracture. The correct diagnosis was confirmed by CT and MRI.

Injuries of the spine most frequently occur in the thoracolumbar regions. This phenomenon is supported by a variety of biomechanical analyses [12]. The thoracolumbar spine is the principal load-bearing structure in the body. This functional role makes the thoracolumbar spine susceptible to injuries. Interestingly, the most common location of butterfly vertebrae is also in this region. McMaster et al. [1] summarized 15 single butterfly vertebrae, and the most common location was T11 in the thoracolumbar region. In our case, the butterfly vertebra was located at T11, and the wedge shape of the vertebra increased the degree of focal kyphosis (Cobb angle = 23°) in this region. These two contributing factors may have increased the risk of incident injury by accentuating the load on the vertebral bodies. In addition, disruption of the PLC in our case also increased spinal instability. Therefore, complete paraplegia occurred in the present case, although there was no fracture of the spine.

In summary, the coexistence of butterfly vertebra and spinal cord injury was reported for the first time. Although butterfly vertebrae may be incidentally detected, it is important to be familiar with their radiographic features to distinguish them from fractures. In this way, a correct diagnosis ultimately can be made, and timely measures can be taken to treat the disease.

## Data Availability

This is a case report of a single patient; in order to protect privacy and respect confidentiality, no part of the raw data has been made available in any public repository. The original operation reports, intraoperative photographs, imaging studies and outpatient clinic records were retained among the medical records of our institution per the normal procedure. All data concerning the case are presented in the manuscript.

## Abbreviations

MRI: Magnetic resonance imaging
CT: Computed tomography
ASIA: American Spinal Injury Association
PLC: Posterior ligament complex
